# Qualitative Evaluation of the Project P.A.T.H.S.: Findings Based on Focus Groups with Student Participants

**DOI:** 10.1100/tsw.2009.59

**Published:** 2009-08-01

**Authors:** Daniel T. L. Shek, Catalina S. M. Ng

**Affiliations:** ^1^ Department of Applied Social Sciences, Hong Kong Polytechnic University, Hong Kong, China; ^2^ Department of Sociology, East China Normal University, Shanghai, China; ^3^ Kiang Wu Nursing College of Macau, Macau, China

**Keywords:** focus groups, adolescents, qualitative evaluation, positive youth development program

## Abstract

This paper reports a qualitative evaluation study conducted to explore the perceptions of students who joined the Tier 1 Program of the Project P.A.T.H.S. A total of 92 students were randomly selected to participate in 10 focus groups, which provided qualitative data for the study. With specific focus on how the informants described the program, the descriptors used were primarily positive; the metaphors named by the informants that could stand for the program were basically positive. Program participants also perceived the program to be beneficial in different psychosocial domains. The present study lends further support to the effectiveness of the Tier 1 Program of the Project P.A.T.H.S. in promoting holistic development in Chinese adolescents.

## INTRODUCTION

With an origin in marketing and social science, focus groups have emerged as a popular tool for generating qualitative data, and are used across a wide variety of disciplines and applied research areas[1]. The mushrooming use of focus groups is evident in the number of citations of focus groups, particularly in health research since the 1980s[2]. In addition, Morgan[3], in his review of online databases, reported that focus groups appeared in 100 academic journal articles per year throughout the decade, and he also provided instances of focus groups being utilized as primary data sources, as supplementary to survey data, and in multimethod studies combined with other methods.

Given the breadth of possible applications of focus groups and their extensive use, much has been written about what focus groups are. A very straightforward definition of focus groups by Morgan and Spanish[4] is “as a qualitative method for gathering data, focus groups bring together several participants to discuss a topic of mutual interest to themselves and the researcher” (p. 253). Similarly, Basch[5] defined the focus groups as “a qualitative research technique used to obtain data about feelings and opinions of a small group of participants about a given problem, experience, service or other phenomenon” (p. 414). Expanding on these definitions, Morgan and Krueger[6] added that a focus group is a “carefully planned series of discussions designed to obtain perceptions on a defined area of interest in a permissive, non-threatening environment" (p. 18). Their definition highlighted that focus groups require thorough planning in advance and the importance of nonthreatening settings, as well as free participation of the participants in the group context. Along the same line, Heary and Hennessy[7] also defined focus groups as thoroughly planned discussions among participants that enable the moderator to obtain individuals' perceptions in a permissive, nonthreatening environment. The definition underscores the importance of the moderator, who is commonly the main instrument to elicit the information in a focus group interview.

As argued by Morgan and Krueger[8], the decision to utilize focus groups in a research study is a decision not to utilize any other possible research methods. In making such a decision, Morgan and Krueger[8] recommended that researchers have to understand the advantages of focus groups. Evidently, one of the principal advantages of focus groups results from the group process and the interaction of group members[9]. Likewise, Twinn[10] stated that the synergism created by the interaction of group members is important to the generation of ideas, which could be difficult to obtain through individual interviews. Focus groups are also advantageous in handling complicated topics in a relatively short period of time. Particularly, the objective of focus groups is not to reach a consensus[11] and data can be gathered at a lower cost than any other qualitative research method[12].

Despite the above advantages, the use of focus groups has been criticized. First, a crucial issue is the heavy reliance placed on the skills of the moderator, particularly in facilitating the group process and interaction, as he/she is expected to probe comments, get answers to questions, and observe nonverbal gestures or responses, all of which may potentially enhance the validity and richness of the data collected[13]. Hence, if the skills of the moderator are problematic, the integrity of the data collected will be substantially impaired. Another criticism of focus groups is that highly sensitive and risky issues can be perceived as threatening, especially when disclosing individuals' perspectives or behaviors in a group context[11].

Interestingly, in spite of their current popularity in different fields of social science, little has been documented about the use of focus groups in program evaluation[7,14]. Ansay et al.[14] highlighted that "although focus groups continue to gain popularity in marketing and social science research, their use in program evaluation has been limited" (p. 310). With reference to 51 promising prevention programs and approaches for at-risk adolescents, the authors found that these programs relied on the sole use of traditional scientific methods, such as random sampling, comparison or control groups, and surveys or other quantitative methods, with statistical significance as the main measure of effectiveness. Another limitation of the literature is that "focus groups appear to have been used quite extensively with populations of black and Hispanic ethnic origins"[15, p. 655] because "this method has been developed for use primarily among Anglo-Celtic populations"[16, p.257]. Because of this limitation, Yelland and Gifford[16] raiseed questions about the appropriateness of focus groups as a data collection tool in cross-cultural research.

There is a remarkable surge of interest in using focus groups in program evaluation in Western countries[13,17]. Nabors and colleagues[18,19] utilized focus groups for an assessment of program needs, strengths, and weaknesses, and to gain ideas for future program development. Recognizing the importance of exploring the contribution of focus groups as a method of qualitative data collection with Chinese populations, Twinn[10,15] conducted several studies in nursing research and concluded in her study[15] that focus groups can be used with Chinese populations. She provided quotations from the data to support her conclusion that the research study with focus group design yielded rich and in-depth data, participants were willing to participate, and that the method is appropriate, with the groups and analysis being conducted in the participants' first language.

To date, little has been documented on the use of focus groups with the Chinese adolescent populations in program evaluation, despite the fact that focus groups are considered to be an effective qualitative data technique that is readily understood by program funders[17]. This paper, therefore, attempts to fill this gap in the literature with specific focus on the Project P.A.T.H.S. To promote holistic adolescent development, the Project P.A.T.H.S. (**P**ositive **A**dolescent **T**raining through **H**olistic **S**ocial Programmes) was initiated by The Hong Kong Jockey Club Charities Trust with an earmarked grant of

HK$400 million. In Hong Kong, results from both published and unpublished studies demonstrated that young people face a number of developmental problems, such as mental health problems like depression[20], abuse of psychotropic substances[21], self-harm[22], adolescent suicide[23], school violence[24], and erosion of family solidarity[20]. Against this background, The Trust invited academics of five universities in Hong Kong to form a research team, with the first author as the Principal Investigator, to develop a multiyear universal positive youth development program to promote holistic adolescent development. There are two tiers of programs (Tier 1 and Tier 2 Programs) in this project. While the Tier 1 Program is a universal program that utilizes a curricula-based approach for all Secondary 1 to 3 students of the participating schools, the Tier 2 Program is provided for at least one-fifth of the students who have greater psychosocial needs.

Since the Project P.A.T.H.S. has a novelty value, and is regarded as a huge project in terms of financial and manpower resources and the number of participating schools in the territory, concerns raised regarding its impact and effectiveness have stimulated rigorous evaluation of the project because, first, it is essential to prove to the program funders that the project is beneficial to students and, second, program implementers (i.e., teachers, social workers, etc.) are only motivated to teach the program that is proved to be effective. Furthermore, reviews of the literature show that there is a pressing need to accumulate research findings on the effectiveness of psychosocial intervention programs. For example, in the Western context, Catalano et al.[25] reported that among the 77 programs under review, only approximately one-third of them were effective, whereas in the Chinese context, Shek et al.[26] highlighted that evidence-based social work practice was very weak in Hong Kong. To provide a comprehensive picture pertaining to the effectiveness of the project, numerous evaluation strategies, including objective outcome evaluation, subjective outcome evaluation, qualitative evaluation based on focus groups, student diaries and in-depth interviews, process evaluation, and interim evaluation are employed. The aforementioned mechanisms provide strong evidence that the Project P.A.T.H.S. is beneficial to students[27,28,29].

Using focus groups to explore participants' perceptions of the program and the perceived program effects is the optimal research technique in the present study as, first, the focus group is particularly useful for "the development of questionnaires, explorations of topics of interest, clarification of content domains, instrument development, outcome evaluations, development and evaluation of training programs…"[30, pp. 190–191). Since the objective of the present study is to explore program participants' perspectives on the program, using focus groups is deemed appropriate. Added to this, focus groups can be used to supplement quantitative methods by facilitating interpretation or by adding depth to responses obtained with quantitative methods[31], and to validate findings[32]. Focus group data can be utilized in conjunction with data from statistical analyses to humanize or "tell the story behind the numbers"[17, p. 251) that we obtained from our numerous evaluation strategies.

In addition to the aforesaid, other strengths of focus group methods make them particularly useful for research with Chinese adolescent populations. Because participants in a focus group setting are accompanied by peers and others who share similar experiences, they feel less pressured and more secure, and are thus willing to share their feelings and experiences[8]. As Umaña-Taylor and Bámaca[33] argued, when there is a lack of trust between participants and researchers, certain research methods (e.g., surveys or questionnaires) can be ineffective. Since adolescents may also be wary of participation for fear of misuse of data, focus groups allow participants to have direct contact with researchers[31]; this contact is crucial for establishing trust between them, and they are more willing to disclose their views or behaviors. Furthermore, in a group setting, participants are not as likely to feel pressured to respond in a certain manner as they might be in a one-to-one dialogue with an adult.

Finally, additional reasons for choosing a focus group format were that focus groups have a high level of face validity[2] as what participants say can be confirmed, reinforced, or contradicted within the group discussion[34], and the results from these groups make sense intuitively and thus they may be more satisfactory to policy makers than results from other methods[35].

Although the focus group is a useful research strategy that can be used to explore the perceptions of the program participants, Webb and Kevern[36] warned that there is a clear need for rigor in the application of focus groups. Similarly, in response to the common problems intrinsic to qualitative studies, Shek et al.[37] argued for the importance of discussing the ideological biases and preoccupations of the researchers in a qualitative evaluation report (Principle 4). As program developers, the authors might have the preoccupation that the implemented program was superb and it was beneficial to the students. Additionally, the researchers may have the tendency to look at positive evidence rather than the negative. Therefore, it is imperative to discuss how such biases were addressed in this study[37].

Several safeguards against the subtle influence of ideological biases and preoccupations were included in the process of the study[37, Principle 5]. First, the researchers were conscious of the existence of ideological preoccupations (e.g., positive youth development programs are beneficial to adolescents), and data collection and data analyses procedures were conducted in a disciplined manner. Second, although the analyses and interpretations were basically carried out by the first author with the assistance of the two research assistants, inter- and intrarater reliability checks on the coding were conducted (Principle 6). Third, multiple researchers and research assistants were involved in the data collection and analysis processes (Principle 7). Fourth, the first author was conscious of the importance and development of audit trails (Principle 9). The tapes, transcriptions, and steps involved in the development of the coding system and interpretations were properly documented and systematically organized.

## METHODS

### Participants

Among the 196 schools participating in the Full Implementation Phase, 80 schools adopted the full program (i.e., 20-h program involving 40 teaching units) and 116 schools adopted the core program (i.e., 10-h program involving 20 teaching units). In the sampling process, eight randomly selected schools that joined the full program and two randomly selected schools that joined the core program were invited to participate in the focus group interviews (i.e., a total of 10 schools). For the consenting schools, the workers randomly selected informants from the program participants to join the focus groups. A total of 92 students joined 10 focus groups of approximately 1-h duration each; the number of informants in each focus group ranged from four to 11 students.

As data collection and analyses in qualitative research are very labor intensive, it is the standard practice to use small samples. As such, the number of focus groups and student participants could be regarded as respectable. Furthermore, the strategy of randomly selecting informants and schools that joined the Tier 1 Program could help to enhance the generalizability of the findings. These arguments satisfy Principle 2 (i.e., justifications for the number and nature of the participants of the study) proposed by Shek et al.[37].

### Procedures

Ten focus groups designed to elicit participant perceptions of the Project P.A.T.H.S. were conducted. The sample was solely Chinese (100%). The researchers and research assistants individually or jointly conducted the focus group interviews. Both parental consent and student consent were obtained prior to the focus group interviews. Since previous studies[e.g., 2,38] emphasized the necessity of careful location selection to conduct focus groups, we decided to choose a place that participants were familiar with so that they felt comfortable when giving opinions[39]. Therefore, we selected the participants' schools, as we thought them to be ideal locations.

During the interviews, the participants were encouraged to express their views about and perceptions of the program. With respect to Principle 3 (i.e., detailed description of the data collection procedures) suggested by Shek et al.[37], the broad interview guide of the focus group interviews is presented in Table 1. The interview questions were designed with reference to both the CIPP model[40] and previous research[41]. In the interviews, the moderators were aware of the importance of adopting an open attitude

**TABLE 1**
**Interview Questions on Product Evaluation**

1. **Evaluation of the General Effectiveness of the Program**
   - Do you feel that the program is beneficial to the development of adolescents?
   - Do you think that the program has helped your development?
   - After participating in the program, do you have any changes? If yes, please specify. (free elicitation)
   - If you feel that you have changed, what do you think are the factors that have promoted such changes?
   - If you have not noticed any changes in yourself, what do you think are the reasons?
2. **Evaluation of the Specific Effectiveness of the Program**
   - Do you think that your participation in the program has affected your school work and grades? Please elaborate your answers.
   - Do you think the program can promote your self-confidence or ability to face the future?
   - Do you think the program can enhance your abilities in different areas in your life?

Optional Questions
   - Do you think the program can promote your spiritual life?
   - Do you think the program can promote your bonding with family, teachers, and friends?
   - Do you think the program can cultivate your compassion and care for others?
   - Do you think the program can promote your participation and care for the society?
   - Do you think the program can promote your sense of responsibility to the society, family, teachers, and peers?
3. **Other Comments**
   - If you are invited to use three descriptors to describe the program, what three descriptors will you use to describe the program?
   - If you are invited to use one incident, object, or feeling (e.g., indigestion, enjoyment, etc.) to describe the program, what metaphors will you use to stand for the program?

to accommodate both positive and negative experiences expressed by the program participants. As the researchers and research assistants conducting the interviews either had training in social group work and/or substantial group work experience, they were conscious of the importance of encouraging the participants to express opinions of a different nature, including both positive and negative views.

## Data Analysis

Due to the dynamic nature of group discussions, it is suggested that focus group data should be analyzed by systematically identifying prominent themes and illustrative statements from the transcripts[2]. Transcription-based analysis is considered to be the most rigorous of the focus group analysis approaches[42]. Thus, the content of the interviews was fully transcribed by student helpers and checked for accuracy by two research assistants. To enhance triangulation in the coding process, two research assistants and the first author were involved in the data analysis of the narratives. Our unit of analysis was a meaningful unit instead of a statement. For instance, the statement that a program was “meaningful and helpful” would be broken down to two meaningful units or attributes, i.e., “meaningful” and “helpful”. Furthermore, descriptions with the same meaning (e.g., “good quality” and “high quality”) were grouped into the same attribute category.

The present coding system was developed after much consideration of the raw data and several preliminary analyses. After initial coding, the positive or negative nature of the codes was determined, with four possibilities (i.e., “positive”, “negative”, “neutral”, and “undecided”). To enhance reliability of the coding of the positive or negative nature of the raw codes, intra- and inter-rater reliability were carried out. In view of the voluminous data collected, qualitative findings on three areas of program evaluation are presented in this paper: (1) descriptors that were used by the informants to describe the program, (2) metaphors (i.e., incidents, objects, or feelings) that were used by the informants to stand for the program, and (3) participants’ perceptions of the benefits of the program to themselves.

## RESULTS

For the descriptors used by the participants to describe the program, there were 144 raw descriptors and they could be further categorized into 41 categories (Table 2). Among these descriptors, 78 (54.2%) of them were coded as positive, which were revealed in the narratives of students: In order to examine the reliability of the coding, the research assistants recoded 20 randomly selected raw descriptors (without knowing the original codes given) at the end of the scoring process. Intrarater agreement percentages calculated from these descriptors were 95 and 100% for the two research assistants, respectively. Finally, these 20 randomly selected descriptors were coded by two colleagues with Master's degrees without knowing the original codes given. Findings indicated that the coded responses corresponded to those of the first author (90 and 100%, respectively).

For the metaphors that were used by the informants that could stand for the program, there were 57 raw "objects" involving 75 related attributes (Table 3). Results demonstrated that 32 metaphors (56.1%) and 43 attributes (57.3%) can be regarded as positive, which was manifested in the following narratives of students:

> *Student: Like a lamp.*
>
> *Moderator: Why?*
>
> *Student: When we have done something wrong it seemed that we were in the dark. The program has taught us many skills and so, it was really like a lamp which led us to the right path.*

To examine the reliability of the coding, the research assistants recoded 20 randomly selected responses without knowing the original codes given at the end of the scoring process. Intrarater agreement percentages calculated from these metaphors were 95 and 100% for the two research assistants, respectively. The metaphors were then coded by two other colleagues with Master's degrees, with high inter-rater agreement with the first author (both of 95%).

Regarding the perceived benefits of the program to the program participants, 234 responses were coded involving 52 attribute categories (Table 4). The findings indicated that 192 responses (82.1%) were coded as positive responses, such as "program meets students' needs", "enhanced interpersonal skills", "identified one's strengths", etc. In order to examine the reliability of the coding, the research assistants recoded 20 randomly selected responses without knowing the original codes given at the end of the scoring process. Intrarater agreement percentages calculated from these responses were 95 and 100%, respectively. The raw benefit categories were coded by, again, two colleagues with Master's degrees without knowing the original codes given. Results demonstrated that inter-rater agreement percentages between these raters and the first author were 95 and 100%, respectively.

## DISCUSSION

In an attempt to explore the perceptions of the program participants pertaining to the qualities and effectiveness of the Tier 1 Program of the Project P.A.T.H.S., this study used focus group methodology to gather qualitative data. Consistent with the findings of Twinn's[15] study, the current study lends further support to the use of focus groups as a good tool for gathering evaluation data with the Chinese populations. Two salient conclusions can be drawn from this study. First, in overall terms, the program was perceived positively from the perspective of the program participants (Tables 2 and 3), although some students perceived the program to be negative, which was not the dominant view. The negative findings are congruent to the observation of Shek[43] that approximately 15% of the participants failed to perceive the program to be effective. Conversely, many participants viewed the program as useful, stimulating, and interesting.

**TABLE 2**
**Categorization of the Descriptors Used by the Participants to Describe the Program**

| Descriptions | Nature of the Response | | | | Total |
|---|---|---|---|---|---|
| | Positive | Neutral | Negative | Undecided | |
| Funny | 12 | | | | 12 |
| Happy | 12 | | | | 12 |
| Surprising | 3 | | | | 3 |
| Learned a lot | 1 | | | | 1 |
| Rich in content | 4 | | | | 4 |
| Meet the needs of students | 2 | | | | 2 |
| Beneficial | 2 | | | | 2 |
| Pride | 2 | | | | 2 |
| Fruitful | 4 | | | | 4 |
| Lively | 1 | | | | 1 |
| Energetic | 1 | | | | 1 |
| Perfect | 1 | | | | 1 |
| Motivating | 1 | | | | 1 |
| Useful | 1 | | | | 1 |
| Good | 4 | | | | 4 |
| Like a teacher | 1 | | | | 1 |
| Attractive | 1 | | | | 1 |
| Relaxing | 10 | | | | 10 |
| Outstanding | 1 | | | | 1 |
| Satisfied | 3 | | | | 3 |
| Confident | 1 | | | | 1 |
| Exciting | 6 | | | | 6 |
| Meaningful | 2 | | | | 2 |
| Not boring | 1 | | | | 1 |
| Serious | 1 | | | | 1 |
| Fair | | 7 | | | 7 |
| To be improved | | 2 | | | 2 |
| Boring | | | 11 | | 11 |
| Meaningless | | | 1 | | 1 |
| Monotonous | | | 3 | | 3 |
| Empty | | | 1 | | 1 |
| Troublesome | | | 4 | | 4 |
| Waste of time | | | 8 | | 8 |
| Nonsense | | | 13 | | 13 |
| Not interactive | | | 1 | | 1 |
| Too relaxing | | | 3 | | 3 |
| Annoying | | | 2 | | 2 |
| Useless | | | 2 | | 2 |
| Unlike a class | | | | 1 | 1 |
| Have no feelings on the program | | | | 3 | 3 |
| Undecided | | | | 4 | 4 |
| **Total Count (N)** | **78** | **9** | **49** | **8** | **144** |
| **Total Count (%)** | **54.17%** | **6.25%** | **34.03%** | **5.56%** | **100%** |

**TABLE 3**
**Categorization of the Metaphors (Incidents, Objects, Feelings, etc.) Used by the Participants to Describe the Program**

| Metaphors | Positive or Negative Nature of the Metaphor | | | | Number of Codes Derived from the Metaphor and Its Nature | | | | |
|---|---|---|---|---|---|---|---|---|---|
| | Positive | Neutral | Negative | Total | Positive | Neutral | Negative | Undecided | Total |
| Shower | 1 | | | 1 | 1 | | | | 1 |
| Rainbow | 1 | | | 1 | 3 | | | | 3 |
| A lesson | 1 | | | 1 | 3 | | | | 3 |
| Refrigerator | 1 | | | 1 | 1 | | | | 1 |
| Fruit | 1 | | | 1 | 1 | | | | 1 |
| Sun | 1 | | | 1 | 1 | | | | 1 |
| Umbrella | 1 | | | 1 | 1 | | | | 1 |
| Stepping stone | 1 | | | 1 | 2 | | | | 2 |
| Egg | 1 | | | 1 | 1 | | | | 1 |
| Human being | 1 | | | 1 | 2 | | | | 2 |
| Zip file | 1 | | | 1 | 1 | | | 1 | 2 |
| Kangaroo | 1 | | | 1 | 2 | | | | 2 |
| Correction fluid | 1 | | | 1 | 1 | | | | 1 |
| Eraser | 1 | | | 1 | 1 | | | | 1 |
| Lucozade | 1 | | | 1 | 1 | | | | 1 |
| Kinder egg | 2 | | | 2 | 2 | | | | 2 |
| An unfair balance | | | 1 | 1 | | | 1 | | 1 |
| Bottle | 1 | | | 1 | 1 | | | | 1 |
| Playing jigsaw puzzle | | 1 | | 1 | | 1 | | | 1 |
| Photo sticker | 1 | | | 1 | 1 | | | | 1 |
| Parenting | | 1 | | 1 | | 1 | | | 1 |
| Tasteless | | | 1 | 1 | | | 2 | | 2 |
| Invisible pen | | | 1 | 1 | | | 1 | | 1 |
| Chicken bone | | 1 | | 1 | | 1 | | | 1 |
| Discipline Master | | | 1 | 1 | | | 2 | | 2 |
| Cicadas | | | 1 | 1 | | | 2 | | 2 |
| Visual Art lesson | 1 | | | 1 | 1 | | | | 1 |
| P.E. lesson | 1 | | | 1 | 2 | | | | 2 |
| Music lesson | 1 | | | 1 | 1 | | | | 1 |
| M&M’s | 1 | | | 1 | 1 | | | | 1 |
| War | | | 1 | 1 | | | 1 | | 1 |
| Telephone | 1 | | | 1 | 1 | | | | 1 |
| Encyclopedia | 1 | | | 1 | 2 | | | | 2 |
| Kung Fu novel | | | 1 | 1 | | | 2 | | 2 |
| Garnish | | 1 | | 1 | | 1 | | | 1 |
| Kindergarten | | 1 | | 1 | | 1 | | | 1 |
| Life philosophy | 1 | | | 1 | 1 | | | | 1 |
| Wet firewood | | | 1 | 1 | | | 1 | | 1 |
| Disappointment | | | 1 | 1 | | | 1 | | 1 |
| Magazine | 1 | | | 1 | 1 | | | | 1 |

*Table 3 continues*

**TABLE 3 (continued)**

| Metaphors | Positive or Negative Nature of the Metaphor | | | | Number of Codes Derived from the Metaphor and Its Nature | | | | |
|---|---|---|---|---|---|---|---|---|---|
| | Positive | Neutral | Negative | Total | Positive | Neutral | Negative | Undecided | Total |
| Bed | | | 1 | 1 | | | 1 | | 1 |
| Lamp | 1 | | | 1 | 1 | | | | 1 |
| Clay | | 1 | | 1 | 2 | | 1 | | 3 |
| Gourd | | 1 | | 1 | | 1 | 1 | | 2 |
| A nod is as good as a wink to a blind horse | | | 1 | 1 | | | 1 | | 1 |
| Cartoon card | | | 1 | 1 | | | 2 | | 2 |
| Rubbish | | | 1 | 1 | | | 1 | | 1 |
| Club | | | 1 | 1 | | | 1 | | 1 |
| Refugee camp | | 1 | | 1 | | 1 | | | 1 |
| Happy Meal | 1 | | | 1 | 1 | | | | 1 |
| Games center | | | 1 | 1 | | | 1 | | 1 |
| Casino | | | 1 | 1 | | | 1 | | 1 |
| Market | | | 1 | 1 | | | 1 | | 1 |
| Main dish | 1 | | | 1 | 1 | | | | 1 |
| Holiday | 1 | | | 1 | 1 | | | | 1 |
| A book | 1 | | | 1 | 1 | | | | 1 |
| **Total Count** | **32** | **8** | **17** | **57** | **43** | **7** | **24** | **1** | **75** |
| **Percentage** | **56.14%** | **14.04%** | **29.82%** | **100%** | **57.33%** | **9.33%** | **32.00%** | **1.33%** | **100%** |

Second, results in Table 4 show that the program had a beneficial effect on the participants, with 82.1% of the responses coded as positive. Generally speaking, benefits in both the personal levels and interpersonal levels were observed. The above observations are generally consistent with the objective outcome evaluation findings of Shek[43] that the students changed in the positive direction in various developmental domains. With reference to the principle of triangulation, the present study and the previous findings suggest that based on both quantitative and qualitative evaluation findings, evidence on the positive effects of the Tier 1 Program on holistic youth development among the program participants is present.

As suggested by Shek et al.[37], it is imperative to consider alternative explanations in the interpretations of qualitative evaluation findings (Principle 10). There are several plausible alternative justifications for the present findings. Initially, the findings can be interpreted in terms of demand characteristics. Nevertheless, this explanation is not likely because the participants were encouraged to express their views freely and negative voices were in fact heard. In addition, since the teachers were not present, there was no need for the students to respond in a socially desirable manner. Another explanation is that the findings were due to selection bias. However, this argument is not strong as the schools and students were randomly selected. Third, the positive findings were due to ideological biases (e.g., self-fulfilling prophecies) of the researchers. Nevertheless, as several safeguards were used to reduce biases in the data collection and analysis processes, this possibility is not high. Finally, it may be argued that the perceived benefits were due to other youth enhancement programs. This argument can be partially dismissed as none of the schools in the present study joined the major youth enhancement programs in Hong Kong, including the Adolescent Health Project and the Understanding the Adolescent Project. Most importantly, participants in the focus group interviews were specifically asked about the program effects of the Project P.A.T.H.S. only.

**TABLE 4**
**Categorization of Responses on the Perceived Benefits of and Things Learned in the Program**

| Category | Subcategory | Responses | Nature of the Response | | | | Total |
|---|---|---|---|---|---|---|---|
| | | | **Positive** | **Neutral** | **Negative** | **Undecided** | |
| Benefits in 2-year Project P.A.T.H.S. learning | — | Positive | 19 | | | | 19 |
| | | Negative | | | 4 | | 4 |
| | | **Subtotal** | **19** | **0** | **4** | **0** | **23** |
| General benefits | — | Program is not beneficial to academic studies | | | 1 | | 1 |
| | | Program meets students' needs | 4 | | | | 4 |
| | | Providing opportunities for students to share | 1 | | | | 1 |
| | | Program is better than normal lesson | 1 | | | | 1 |
| | | Positive comments | 32 | | | | 32 |
| | | Neutral comments | | 1 | | | 1 |
| | | Negative comments | | | 29 | | 29 |
| | | Undecided | | | | 7 | 7 |
| | | **Subtotal** | **38** | **1** | **30** | **7** | **76** |
| Societal level | — | Increased awareness of different social issues | 2 | | | | 2 |
| | | **Subtotal** | **2** | **0** | **0** | **0** | **2** |
| Familial level | — | Enhanced family relationship | 1 | | | | 1 |
| | | **Subtotal** | **1** | **0** | **0** | **0** | **1** |
| Interpersonal level | General interpersonal competence | Enhanced peer relationship | 5 | | | | 5 |
| | | Enhanced teacher-student relationship | 4 | | | | 4 |
| | | Strengthened connection with healthy adults | 1 | | | | 1 |
| | | Enhanced interpersonal skills | 11 | | | | 11 |
| | | **Subtotal** | **21** | **0** | **0** | **0** | **21** |
| | Specific interpersonal competence | Become a good listener | 2 | | | | 2 |
| | | Enhanced communication skills | 3 | | | | 3 |
| | | Take care of others | 1 | | | | 1 |
| | | Better understanding of others | 5 | | | | 5 |
| | | Respect others | 1 | | | | 1 |
| | | Enhanced conflict resolution skills | 1 | | | | 1 |
| | | Accept others' opinions | 2 | | | | 2 |
| | | Empathy | 2 | | | | 2 |
| | | Make apology | 1 | | | | 1 |
| | | **Subtotal** | **18** | **0** | **0** | **0** | **18** |

*Table 4 continues*

**TABLE 4 (continued)**

| Category | Subcategory | Responses | Nature of the Response | | | | Total |
|---|---|---|---|---|---|---|---|
| | | | Positive | Neutral | Negative | Undecided | |
| Personal level | Positive self-image | Personal growth | 4 | | | | 4 |
| | | Enhanced self-understanding | 7 | | | | 7 |
| | | Identified one's strengths | 1 | | | | 1 |
| | | Gained wisdom | 2 | | | | 2 |
| | | Have little personal changes | 1 | | | | 1 |
| | | Enhanced self-confidence | 10 | | | | 10 |
| | | Self-determination | 1 | | | | 1 |
| | | **Subtotal** | **26** | **0** | **0** | **0** | **26** |
| | Emotional competence | Good temper | 2 | | | | 2 |
| | | Emotional management | 11 | | | | 11 |
| | | **Subtotal** | **13** | **0** | **0** | **0** | **13** |
| | Cognitive competence | Enhanced self-reflection | 4 | | | | 4 |
| | | Open minded | 1 | | | | 1 |
| | | Be optimistic | 1 | | | | 1 |
| | | Positive thinking | 4 | | | | 4 |
| | | Critical thinking | 10 | | | | 10 |
| | | Distinguish right/wrong | 2 | | | | 2 |
| | | **Subtotal** | **22** | **0** | **0** | **0** | **22** |
| | Behavioral competence | Enhanced problem-solving skills | 4 | | | | 4 |
| | | Take initiative | 3 | | | | 3 |
| | | Enhanced presentation skills | 6 | | | | 6 |
| | | Put more effort on studies | 1 | | | | 1 |
| | | More concentrated in the lessons | 2 | | | | 2 |
| | | Preparing for the future | 2 | | | | 2 |
| | | Goal setting | 4 | | | | 4 |
| | | Eliminated bad habits | 1 | | | | 1 |
| | | Work seriously | 1 | | | | 1 |
| | | How to reject others | 1 | | | | 1 |
| | | Willing to try things | 1 | | | | 1 |
| | | How to deal with adversity | 6 | | | | 6 |
| | | **Subtotal** | **32** | **0** | **0** | **0** | **32** |
| | | **Total Count** | **192** | **1** | **34** | **7** | **234** |
| | | **Percentage** | **82.1%** | **0.4%** | **14.5%** | **3%** | **100%** |

As argued by Shek et al.[37], the authors should discuss the limitations of the qualitative evaluation studies conducted (Principle 12); the limitations of the study are stated below. Primarily, several general limitations involved in focus groups are worth noting. First, focus groups provide descriptions about perceptions of the program participants and they are not useful for testing hypotheses in the traditional experimental design. Second, although the group interaction is generally seen as an advantage of focus groups, Lewis[44] argued that there is always the possibility that intimidation within the group setting may inhibit interaction. Another obstacle not encountered in individual interviews is scheduling a time and location convenient to all participants. Further, caution must also be exercised as the quality of the findings is tied to the skills of the moderator[7]. Regarding the second and third limitation, the use of experienced moderators in this study could minimize the problems.

There are other specific limitations of the present study. First, focus group data were only collected at one time point. In addition to the one-shot focus group interviews, it would be illuminating if regular and ongoing qualitative evaluation data could be collected. Next, although observation data have been collected[37], the inclusion of other qualitative evaluation strategies, such as in-depth individual interviews, would be helpful to further understand the subjective experiences of the program participants. Finally, although 11 principles proposed by Shek et al.[37] were upheld in the present study, peer checking and member checking (Principle 8) were not carried out due to time and manpower constraints. Despite these limitations, the present study provides pioneering qualitative evaluation findings supporting the positive nature of the Project P.A.T.H.S. and its effectiveness in promoting holistic youth development among Chinese adolescents in Hong Kong. The current study extends the published literature by using focus group methodology with Chinese populations in program evaluation, which has been under-reported.

## ACKNOWLEDGMENTS

The preparation for this paper and the Project P.A.T.H.S. were financially supported by The Hong Kong Jockey Club Charities Trust.
